# Degradation of TRPML1 in Neurons Reduces Neuron Survival in Transient Global Cerebral Ischemia

**DOI:** 10.1155/2018/4612727

**Published:** 2018-12-18

**Authors:** Yang Wang, Shao-wei Jiang, Xuan Liu, Lei Niu, Xiao-li Ge, Jin-cheng Zhang, Hai-rong Wang, Ai-hua Fei, Cheng-jin Gao, Shu-ming Pan

**Affiliations:** ^1^Department of Emergency, Xinhua Hospital, Shanghai Jiao Tong University School of Medicine, Shanghai 200092, China; ^2^Department of Critical Care Medicine, Zhongshan Hospital, Fudan University, Shanghai 200032, China

## Abstract

Postcardiac arrest syndrome yields poor neurological outcomes, but the mechanisms underlying this condition remain poorly understood. Autophagy plays an important role in neuronal apoptosis induced by ischemia. However, whether autophagy is involved in neuron apoptosis induced by cardiac arrest has been less studied. This study found that TRPML1 participates in cerebral ischemic reperfusion injury. Primary neurons were isolated and treated with mucolipin synthetic agonist 1 (ML-SA1), as well as infected with the recombinant lentivirus TRPML1 overexpression vector *in vitro*. ML-SA1 was delivered intracerebroventricularly in transient global ischemia model. Protein expression levels were determined by western blot. Neurological deficit score and the infarct volume were analyzed for the detection of neuronal damage. We found that TRPML1 was significantly downregulated *in vivo* and *in vitro* ischemic reperfusion model. We also observed that TRPML1 overexpression or treatment with the ML-SA1 attenuated neuronal death in primary neurons and ameliorated neurological dysfunction *in vivo.* Our findings suggested that autophagy and apoptosis were activated after transient global ischemia. Administration of ML-SA1 before transient global ischemia ameliorated neurological dysfunction possibly through the promotion of autophagy and the inhibition of apoptosis.

## 1. Introduction

Neurological deficits after resuscitation contribute to high rates of mortality among patients after cardiac arrest [1–3]. Although recent advances in cardiopulmonary resuscitation techniques have significantly increased the rate of return of spontaneous circulation, few cardiac arrest victims achieve meaningful neurologic recovery [4–6]. Transient global cerebral ischemia (TGCI) is a clinical outcome related to cardiac arrest and other situations that deprive the oxygen and glucose in the brain during a short period. Severer neurologic deficits develop in the rare cases of survival from an unwitnessed cardiac arrest than in patients who have survived a witnessed cardiac arrest [7]. Therefore, the latent and persistent neurologic injuries that accompany TGCI may be the greatest obstacle to a full recovery from cardiac arrest, resulting in a broad range of neurological dysfunction [8–10].

Autophagy plays an important role in neuronal apoptosis induced by ischemia. As reported, autophagy protected the neurons from apoptosis both in the oxygen-glucose deprivation (OGD) model and the mouse cerebral ischemia model, via inhibiting neuronal apoptosis [11, 12]. Additionally, pharmacological induction of autophagy contributed to the neuron survival by suppressing apoptosis in the rat middle cerebral artery occlusion (MCAO) stoke model [13]. Furthermore, Wang et al. have demonstrated that deletion of *β*-arrestin-1, a vital scaffolding protein interacted with beclin-1 and Vps34 forming a proautophagic complex in neurons, hampered autophagosome formation and enhanced neural apoptosis [14]. However, whether autophagy is involved in neuron apoptosis induced by cardiac arrest has been less studied.

Transient receptor potential mucolipin-1 (TRPML1), also named MCOLN1, is widely expressed in mammalian cell lysosomes or in the endosome membrane [15] and is the main channel for lysosome Ca^2+^ release and the key regulator for lysosomal storage and transportation [16, 17]. In addition, TRPML1 mutations can not only affect the activation of transcription factor EB (TFEB) but also the regulation of oxidative stress. Li et al. [18] determined TRPML1 is required for the activation of mammalian target of rapamycin complex 1 (mTORC1), which plays a fundamental role in autophagy regulation. TRPML1 has been also supposed to play a dual role in autophagosome membrane fusion and fission in the late endocytic pathways [15]. Further, impairment of autophagy has been reported in TRPML1^−/−^ cells [19, 20]. Recently, Zhang et al. [17] showed TRPML1 could induce autophagy in a mTOR-independent way under stress conditions. Thus, TRPML1 is crucial for the regulation of autophagy [17, 21, 22].

However, the role of TRPML1 in cardiac arrest-induced ischemia is largely unknown. In this study, we hypothesize that TRPML1 is involved in ischemic reperfusion brain injury. Additionally, we aimed to assess the possible interaction between TRPML1 and neuronal injury. Furthermore, we attempt to describe a comprehensive mechanism of the effect of TRPML1 on ischemic reperfusion brain injury.

## 2. Materials and Methods

### 2.1. Animals

All animal experiments were performed in accordance with Xinhua Hospital Affiliated to Shanghai Jiao Tong University School of Medicine (Shanghai, China) guidelines for the use of experimental animals and the experimental protocols were approved by the Institutional Authority for Laboratory Animal Care of Xinhua Hospital Affiliated to Shanghai Jiao Tong University School of Medicine. All animal experiments were strictly followed with the guidelines of animal welfare in Xinhua Hospital Affiliated to Shanghai Jiao Tong University School of Medicine. The C57BL/6 mice were raised under standard laboratory conditions with food and water freely available. Humidity and temperature were kept constant at 60 ± 5% and 24 ± 2°C, respectively. Lights were on between 7:00 am and 7:00 pm.

### 2.2. Surgery for Mouse Models of Transient Global Cerebral Ischemia

The male C57BL/6 mice (8–10 weeks) were randomly assigned to the indicated groups and anesthetized with 4% isoflurane in 70% N_2_O and 30% O_2_. The transient bilateral common carotid artery occlusion (tBCCAO) surgery was performed as described previously [23] with minor modifications. Briefly, tBCCAO was initiated by occluding both common carotid arteries with microclips. After 40 min of ischemia, the clips were removed from both the arteries to allow for recirculation of the blood. The body temperature of mice was maintained at 37°C, and cerebral blood flow was examined during ischemia.

### 2.3. Triphenyl Tetrazolium Chloride (TTC) Staining

The mice were sacrificed and the brains were then dissected. The brain slices (2–3 mm) were made coronally and incubated with 2% TTC in 0.9% saline at 37°C for 15 min. After that, the TTC solution was replaced with PBS and the brain slices were kept in 4% PFA. Infarct size was measured by an observer blinded to the experimental group assignment using the Image-Pro Plus. The infarct volume for each brain was calculated as IV% = (total volume of brain—normal volume of brain)/total volume of brain.

### 2.4. Neurological Deficit Score

The neurological deficit was determined using a 5-point-scale standard in a blinded fashion modified from described previously [24]: 0, no observable deficit; 1, torso flexion; 2, convulsion; 3, no spontaneous movement; 4, no forced movement; 5, death.

### 2.5. ML-SA1 Infusions

For ML-SA1 infusions, the reagent-infusion tubes were inserted into the lateral ventricles and fixed with the acrylic and dental cement. ML-SA1 was delivered intracerebroventricularly at a rate of 0.3 *μ*l/min after 10-day recovery period. The coordinates of the lateral ventricles are AP −0.3 mm, ML ±1.0 mm, and DV −2.2 mm from bregma.

### 2.6. Isolation of the Adult Neurons and Astrocytes

The isolated mouse hippocampus or cortex was digested into cell suspension with Adult Brain Dissociation Kit (Miltenyi Biotec) following the vender protocol. The neurons or astrocytes were sorted by Neuron Isolation Kit (Miltenyi Biotec) or Anti-GLAST (ACSA-1) MicroBead Kit (Miltenyi Biotec), respectively, according to the manufacturer's instructions.

### 2.7. Cell Culture, Transfection

For hippocampal neuron culture, mouse hippocampus was isolated in cold PBS and then digested in 0.25% trypsin for 6 min at 37°C. After digestion termination by fetal bovine serum (FBS, Invitrogen), the cells were filtered through a 70 *μ*m nylon strainer and then centrifuged at 800 rpm for 5 min. The cell pellets were resuspended in Dulbecco's modified Eagle's medium (DMEM, Invitrogen) supplemented with 20% FBS for 4 hr culture, and then the medium was replaced with Neurobasal (Invitrogen). After 3 days of culture, uridine (17.5 *μ*g/ml) and 5-fluoro-2′-deoxyuridine (7.5 *μ*g/ml) were added to the medium to limit the glia growth. For astrocyte culture, cerebral cortex was obtained from C57BL/6 mice between postnatal 1 and 3 days (P1-3) and cultured in DMEM supplemented with 10% FBS.

To package the TRPML1 lentivirus, the sequence containing TRPML1 cDNA (NCBI reference sequence: NM_053177.1) was cloned into pLenti-CMV-EGFP plasmid. The titers of lentivirus particles were between 5 × 10^8^ and 1 × 10^9^ units/ml. The hippocampal neuron culture was used for drug treatment after 48 hrs virus transfection.

### 2.8. OGD and Drug Treatment

For OGD, the culture medium of hippocampal neurons and astrocyte was replaced with glucose-free Earle's balanced salt solution purged by 5% CO2 and 95% N2 for 10 minutes. The cells were then kept with 5% CO2 and 95% N2 for another 0.5 or 1 hrs. ML-SA1 (10 *μ*M), calpeptin (10 *μ*M), DEVD (10 *μ*M), or MG-132 (5 *μ*M) was added into the culture medium before OGD.

### 2.9. MTT Assay

After OGD, the medium of the primary cultured hippocampal neurons or astrocytes was replaced with 2 mg/ml MTT in PBS for 6 hr incubation at 37°C. After addition of DMSO, the plate was gently rotated for 20 minutes to solubilize the eventually formed formazan crystals. Finally, the absorbance value of 570 nm was measured using a microplate reader to determine the optic density.

### 2.10. RNA Isolation, Reverse Transcription, and qRT-PCR

The primary cultured hippocampal neurons were harvested and lysed in TriZol (Sigma), and then the total RNA was extracted. 1 *μ*g RNA was reverse-transcripted into cDNA using M-MLV reverse transcriptase following by the procedure: 10 min, 25°C; 60 min, 42°C; and10 min, 70°C. The expression levels of TRPML1 and *β*-actin were quantified by Step One Plus Real-Time PCR System (ABI) with the two-step protocol (95°C, 10 min; 95°C, 10 s, and 60°C, 1 min for 40 cycles; 95°C, 15 s; 60°C, 1 min.).

Primers were listed as follows:

TRPML1.

Forward 5′-CAAGATCTTGGTGGTCACTGTGCAG-3′

Reverse 5′-GGTTGCTGAGCCCAAAGAGAATGAG-3′

*β*-Actin

Forward 5′-GGCTGTATTCCCCTCCATCG-3′

Reverse 5′-CCAGTTGGTAACAATGCCATGT-3′

### 2.11. Western Blot Analysis

The primary cultured hippocampal neurons/astrocytes and neurons/astrocytes sorted from the mouse brain tissues were harvested and lysed in 2% SDS lysis buffer. The total protein were T electrophoresed on SDS-8/12%PAGE and then transferred to polyvinyl difluoridine membranes. After blocking with 5% milk in PBS, the protein was incubated with the indicated antibody (TRPML1, 1 : 100; HA, 1 : 2000; LC3, 1 : 1000; NeuN, 1 : 1000; GFAP, 1 : 1000; cleaved caspase3, 1 : 500; and *β*-actin 1 : 5000) overnight at 4°C. The next day, the secondary antibodies (1 : 5000) were added after washing with PBS. The protein bands were visualized by Tanon 5200 (Tanon, China) using an ECL western blotting substrate kit, and the band density was analyzed by ImageJ.

#### 2.11.1. Flow Cytometry

Apoptosis was analyzed using annexin V-FITC Apoptosis Detection Kit (BD Biosciences Pharmingen, San Diego, CA) and BD Biosciences FACSCalibur flow cytometry. Briefly, a total of 5^∗^10^5^ cells were washed twice with cold PBS and resuspended with binding buffer and then stained with annexin V-FITC and propidium iodide (PI) for 15 min in the dark. Flow cytometry was analyzed using BD Biosciences FACSCalibur within 1 hour.

### 2.12. Drugs and Antibodies

Antibodies were used as follows: TRPML1 from Alomone Labs (acc-081), GFAP from Sigma (G3893), NeuN from Abcam (ab177487), HA from Sigma (H6908), LC3 from Abcam (ab192890), beclin-1 from Cell Signaling Technology (#3495), p62 from Proteintech (18420-1-AP), cleaved caspase3 from Cell Signaling Technology (#9661), and *β*-actin from Abcam (ab8227). Drugs were used as follows: ML-SA1 from Abcam (ab144622), MG-132 from Medchemexpress (HY-13259), DEVD from Medchemexpress (HY-12466), and calpeptin from Abcam (ab120804).

### 2.13. Statistical Analysis

All the data were presented as means ± SEM from at least three independent experiments. Two-tailed Student's *t*-test was performed to compare the differences between two groups and one-way ANOVA with Tukey's post hoc test performed to compare multiple groups using GraphPad Prism 5. Differences were considered to be significant when a *P* value is less than 0.05.

## 3. Results

### 3.1. TRPML1 Is Specifically Downregulated in Hippocampal Neurons through Proteasome in Ischemia

**Figure 1:**
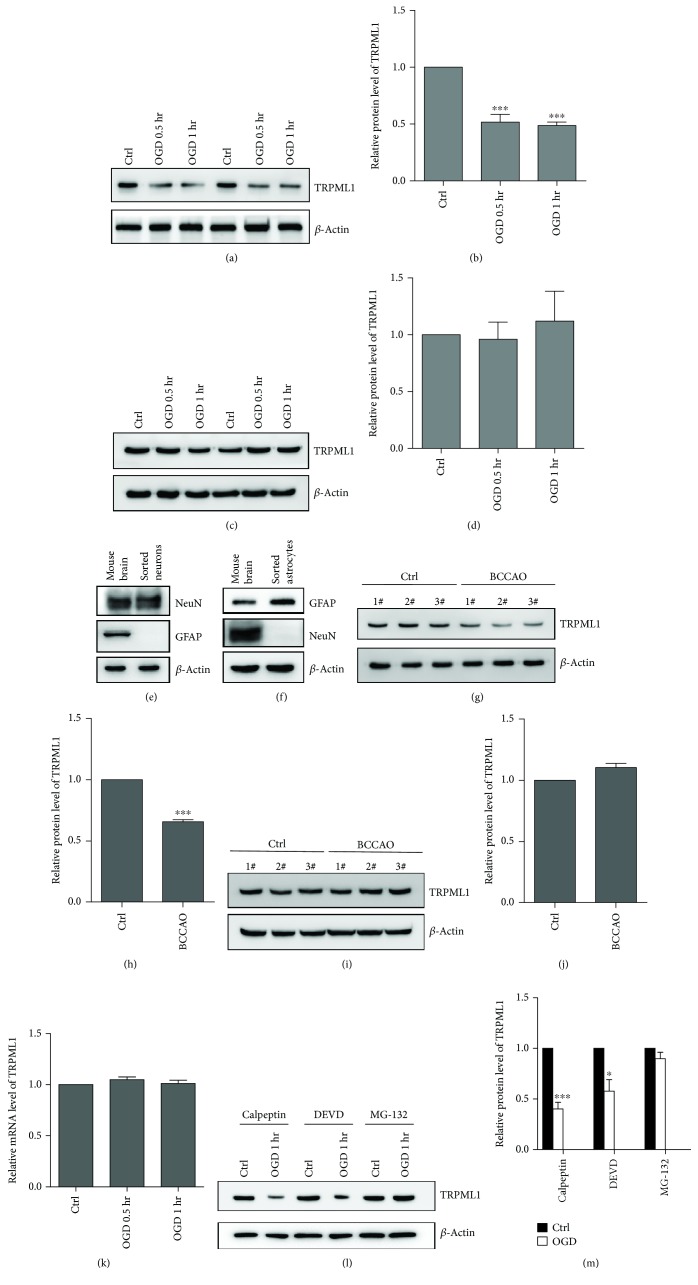
Proteasome-mediated degradation of TRPML1 of hippocampal neurons in ischemia. (a–d) Representative immunoblots of TRPML1 from cultured hippocampal neurons (a, *n* = 4) or astrocytes (c, *n* = 4) 0.5 or 1 hrs after OGD. (b) or (d) was the statistics for (a) or (c). (e, f) Immunoblots of analysis of the indicated marker expression in sorted neurons (e) and astrocytes (f) (GFAP: astrocyte marker, NeuN: neuron marker). (g–j) Representative immunoblots of TRPML1 from sorted neurons (g, *n* = 9) or astrocytes (i, *n* = 9) 40 min after tBCCAo. (h) or (j) was the statistics for (g) or (i). (k) Quantitative PCR analysis of TRPML1 mRNA levels in hippocampal neurons 1 hr after OGD (*n* = 5). (l, m) Representative immunoblots of TRPML1 from cultured hippocampal neurons (*n* = 5) incubated with/without calpeptin (10 *μ*M), DEVD (10 *μ*M), or MG132 (5 *μ*M). (m) was the statistics for (l). *β*-Actin serves as a loading control. All data were presented as mean ± SEM. Comparisons between groups for statistical significance were performed with one-way analysis of variance (ANOVA) with Tukey's post hoc test (b, d, and k), Student's *t*-test, two tailed (h, j), or two-way ANOVA with Bonferroni post hoc test (m). ^∗^*P* < 0.05, ^∗∗∗^*P* < 0.001 versus Ctrl.

To investigate the role of TRPML1 in neuronal survival of ischemia reperfusion, the primary cultured hippocampal neurons were initially deprived of oxygen and glucose to mimic in vivo ischemia. As shown in Figures 1(a)–1(b), the protein expression of TRPML1 was greatly downregulated in the primary cultured hippocampal neurons 30 min after OGD and maintained its reduced level to one hour after OGD. In contrast, the protein level of TRPML1 in astrocytes was not changed 30 min or one hour after OGD (Figures 1(c)–1(d)).

We next studied the TRPML1 expression of hippocampal neurons in the tBCCAo model. Hippocampal neurons or astrocytes were isolated from the adult mouse hippocampus or total brain, respectively, and then verified by the expression of hexaribonucleotide binding protein-3 (NeuN), a marker for neurons and glial fibrillary acid protein (GFAP), a marker for astrocytes using western blotting (Figures 1(e)–1(f)). Consistent with the results from primary cultured hippocampal neurons, we found the neuronal TRPML1 protein was also downregulated in the mouse hippocampus after tBCCAo (Figures 1(g)–1(h)), while the protein expression of TRPML1 in astrocytes was not changed (Figures 1(i)–1(j)). Taken together, these findings suggest that TRPML1 is specifically reduced in hippocampal neurons in ischemia reperfusion.

To further explore the mechanism underlying the downregulation of TRPML1 of hippocampal neurons in ischemia reperfusion, we examined the TRPML1 mRNA level by quantitative real-time PCR (qRT-PCR). As shown in Figure 1(k), the mRNA level of TRPML1 in the primary cultured hippocampal neurons was not changed both 30 min and one hour after OGD. These results indicated that the downregulation of TRPML1 was probably regulated at its posttranscriptional level. As reported, protease calpain or caspase-3 plays an important role in protein cleavage and stability [25–27]. However, administration of calpeptin, the inhibitor of calpain or DEVD, the caspase-3 inhibitor did not prevent the TRPML1 degradation in hippocampal neurons (Figures 1(l)–1(m)). Notably, incubation of MG-132, the inhibitor of proteasome, significantly inhibited the reduction of TRPML1 level (Figures 1(l)–1(m)). Altogether, these observations indicated that proteasome mediates the TRPML1 degradation of hippocampal neurons in ischemia reperfusion.

### 3.2. TRPML1 Reduces the Hippocampal Neuron Death after OGD

**Figure 2:**
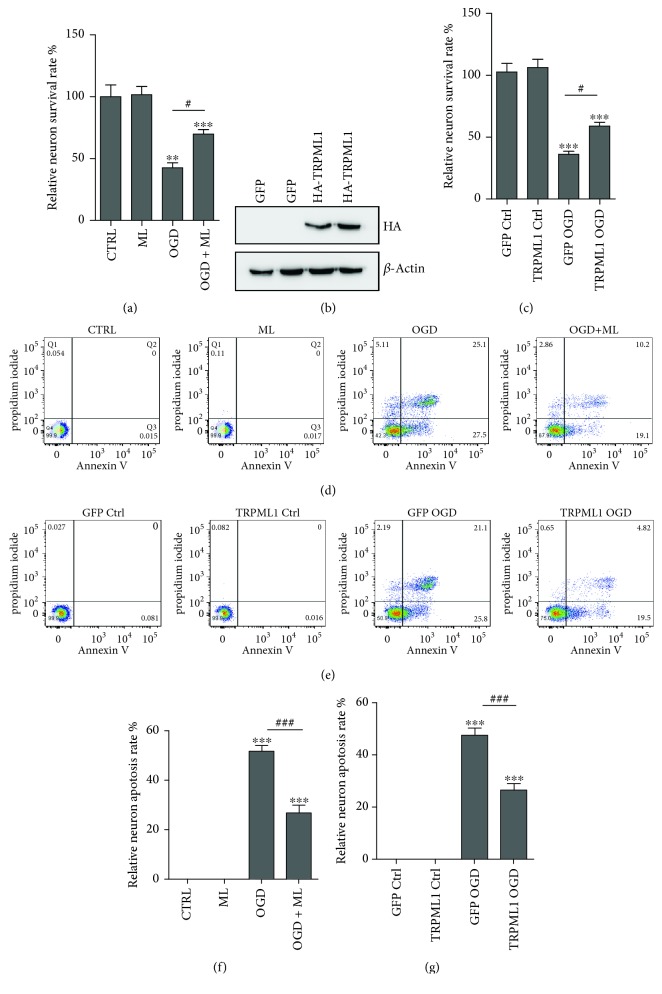
Reduction of neuron survival in OGD by TRPML1 degradation. (a) Hippocampal neuron survival rate determined by MTT assay after 3 hrs OGD in the preincubation with ML-SA1 (10 *μ*M, *n* = 4). (b) Immunoblot analysis of HA-TRPML1 expression in hippocampal neuron. (c) The survival rate of hippocampal neuron transfected with TRPML1 lentivirus determined by MTT assay after 3 hrs OGD (*n* = 5). *β*-Actin serves as a loading control. (d, f) Hippocampal neuron apoptosis was analyzed by flow cytometry 3 hrs OGD in the preincubation with ML-SA1 (10 *μ*M, *n* = 4). (e, g) The apoptosis of hippocampal neuron transfected with TRPML1 lentivirus was determined by flow cytometry after 3 hrs OGD (*n* = 4). All data were presented as mean ± SEM. All comparisons between groups for statistical significance were performed with one-way analysis of variance (ANOVA) with Tukey's post hoc test. ^∗∗^*P* < 0.01, ^∗∗∗^*P* < 0.001 versus Ctrl. ^#^*P* < 0.05 versus OGD.

To study the role of TRPML1 degradation in ischemia reperfusion, we next measured the hippocampal neuron survival by MTT assay after OGD. The results in Figure 2(a) showed that the death rate of hippocampal neuron after OGD was about 60%. Incubation of ML-SA1, the TRPML1 agonist, significantly improved the neuron survival after OGD (Figure 2(a)). Furthermore, we engineered a hemagglutinin- (HA-) tagged TRPML1-2A-green fluorescent protein (GFP) lentivirus, which was well expressed in hippocampal neurons (Figure 2(b)). In accordance with the result of ML-SA1 treatment, the enhanced expression of TRPML1 also largely reduced the hippocampal neuron death after OGD (Figure 2(c)).

To further explore the apoptosis rate of hippocampal neurons in ischemia reperfusion, we examined the apoptosis rate by flow cytometry. The results in Figures 2(d)–2(g) showed the apoptosis rate was greatly increased after OGD. In addition, incubation of ML-SA1 significantly improved the neuron survival after OGD (Figures 2(d) and 2(f)). In accordance with the result of ML-SA1 treatment, the enhanced expression of TRPML1 also largely reduced the hippocampal neuron death after OGD (Figures 2(e) and 2(g)).

### 3.3. TRPML1 Activation Promotes Autophagy and Inhibits Apoptosis

**Figure 3:**
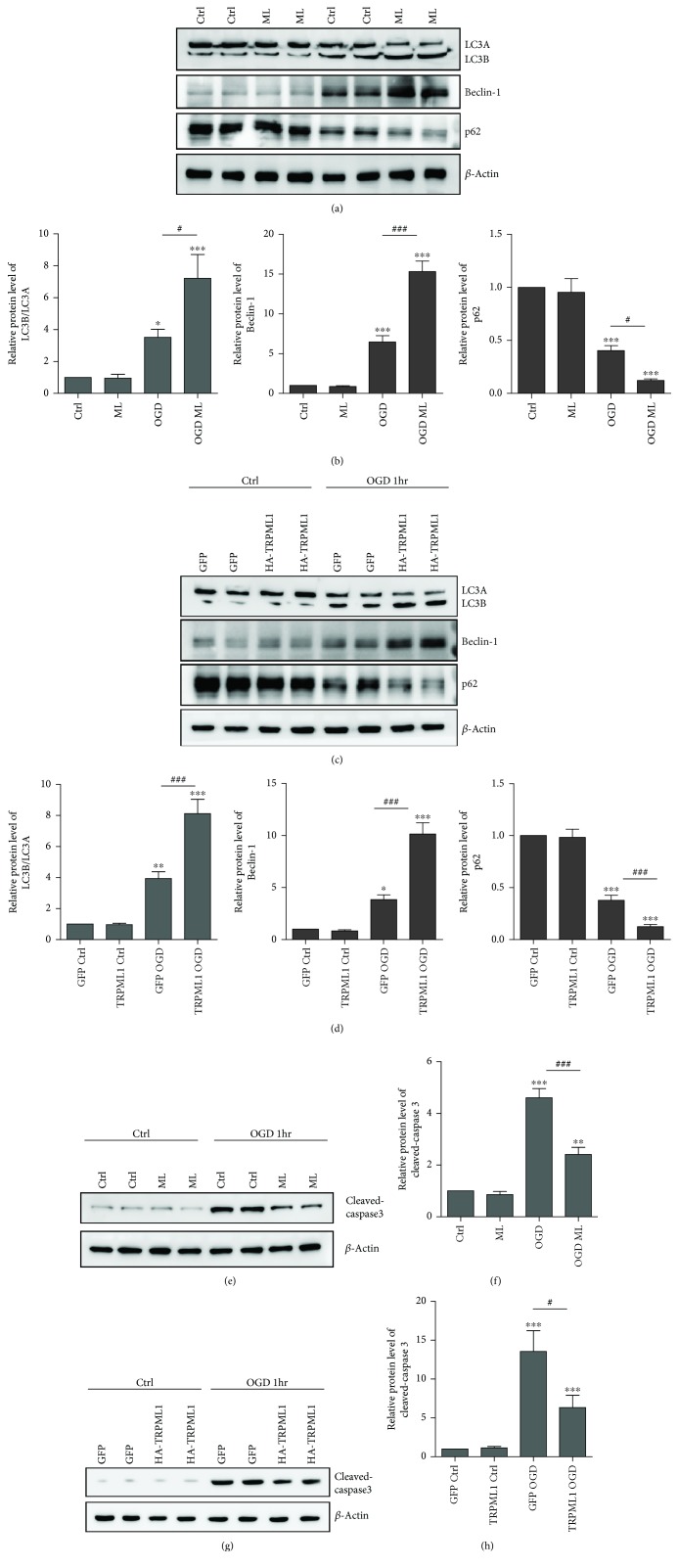
Autophagy enhancement and apoptosis inhibition by TRPML1 activation. (a–d) Representative immunoblots of beclin-1, LC3, and p62 from cultured hippocampal neurons preincubated with 10 *μ*M ML-SA1 (a, *n* = 6) or transfected with TRPML1 lentivirus (c, *n* = 6) 1 hr after OGD. (b) or (d) was the statistics for (a) or (c). (e–h) Representative immunoblots of cleaved caspase3 from cultured hippocampal neurons preincubated with 10 *μ*M ML-SA1 (e, *n* = 6) or transfected with TRPML1 lentivirus (g, *n* = 6) 1 hr after OGD. (f) or (h) was the statistics for (e) or (g). *β*-Actin serves as a loading control. All data were presented as mean ± SEM. All comparisons between groups for statistical significance were performed with one-way analysis of variance (ANOVA) with Tukey's post hoc test. ^∗^*P* < 0.05,^∗∗^*P* < 0.01, ^∗∗∗^*P* < 0.001 versus Ctrl. ^#^*P* < 0.05, ^###^*P* < 0.001 versus OGD.

The findings above suggested that TRPML1 degradation in ischemia reperfusion resulted in the death of hippocampal neurons. To explore the mechanism underlying it, we then studied the autophagy levels in the hippocampal neuron after TRPML1 degradation. As shown in Figures 3(a)–3(b), the expression level of autophagy markers beclin-1 and LC3B was greatly increased after OGD. Moreover, incubation of ML-SA1 further potentiated the beclin-1 and LC3B expression in the hippocampal neurons (Figures 3(a)–3(b)). Additionally, the enhancement of TRPML1 expression also contributes to the elevation of beclin-1 and LC3B level in the hippocampal neurons (Figures 3(c)–3(d)). Analysis of p62 expression demonstrated that incubation of ML-SA1 or TRPML1 overexpression in OGD decreased the levels of p62. These results indicated that TRPML1 activation potentiated the autophagy level in ischemia reperfusion.

It has been reported that enhancement of autophagy level could prevent neuronal loss from apoptosis in ischemia [13]. To further study whether TRPML1-regulated autophagy could affect the neuronal loss, we examined the expression level of cleaved caspase-3 in the hippocampal neurons. The results in Figures 3(e)–3(f) showed that OGD induced the production of cleaved caspase-3 in the hippocampal neurons, which was remarkably inhibited by ML-SA1 treatment. Consistently, exogenous expression of TRPML1 also reduced the elevated expression of cleaved caspase-3 induced by OGD. As talked above, the results of flow cytometry after OGD showed the ameliorated apoptosis rate of neurons by either ML-SA1 treament or lentivirus overexpression (Figures 2(d)–2(e)). Taken together, downregulation of TRPML1 level in the hippocampal neurons inhibits autophagy and contributes to cell apoptosis.

### 3.4. TRPML1 Activation Prevents Brain Damage and Improves the Survival Rate in Transient GCI

**Figure 4:**
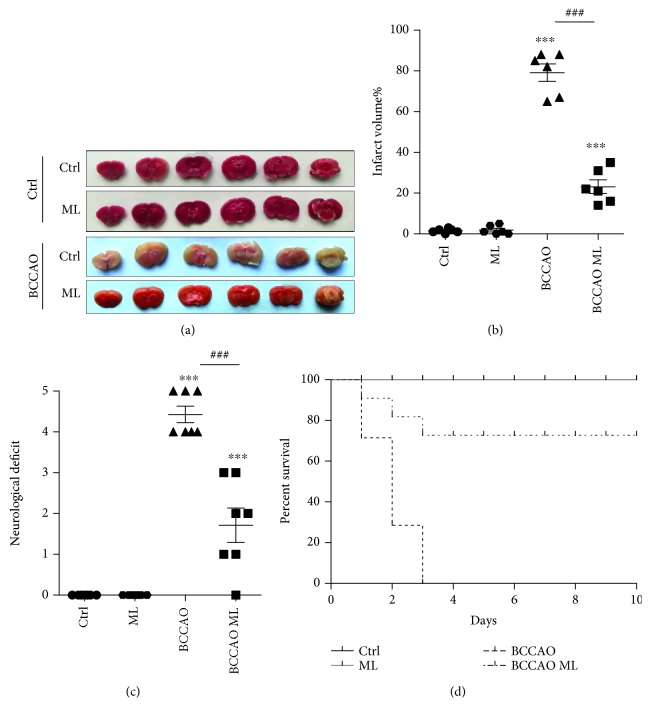
Reduction of the infarct volumes and mortality by TRPML1 activation. (a, b) Infarct volumes of mice infusion with or without 5 *μ*M ML-SA1after 12 hrs reperfusion (*n* = 6). (b) was the statistics for (a). (c) Evaluation of neurological deficit after 24 hrs reperfusion was evaluated in mice infusion with or without 5 *μ*M ML-SA1 (*n* = 7). (d) Evaluation of the survival rate in mice infusion with or without 5 *μ*M ML-SA1 after tBCCAO (*n* = 8). All data were presented as mean ± SEM. All comparisons between groups for statistical significance were performed with one-way analysis of variance (ANOVA) with Tukey's post hoc test. ^∗∗∗^*P* < 0.001 versus Ctrl. ^###^*P* < 0.001 versus OGD.

To further explore the role of TRPML1 in the tBCCAO model, we delivered ML-SA1 into lateral ventricles of the mice. As shown by TTC staining in Figures 4(a) and 4(b), tBCCAO greatly induced the infarct volumes compared with control, while infusion of ML-SA1 significantly reduced the infarct volumes. Moreover, we measured the neurological deficits 24 hrs after tBCCAO and the mouse survival rate ten days after tBCCAO. Notably, ML-SA1 delivery remarkably improved the mouse behavior in neurological deficit score and increased the mouse survival rate (Figures 4(c)–4(d)). Collectively, our results above suggested that enhancement of TRPML1 activity in ischemia reperfusion protected the neurons from damage and reduced the mortality.

## 4. Discussion

Here, we found degradation of TRPML1 reduced neuron survival after ischemic reperfusion injury.

Several lines of evidence support this conclusion. First, the neuron protein level of TRPML1 was downregulated after ischemic reperfusion injury both in vivo and vitro. Furthermore, the decreased TRPML1 protein level could be inhibited by proteasome inhibitor MG132. Second, upregulating TRPML1 by both agonist ML-SA1 and overexpression lentivirus could increase survival rate in neurons. Third, we found autophagy was enhanced and apoptosis was suppressed after TRPML1 activation. Lastly, upregulating TRPML1 in TGI mice could prevent brain damage, ameliorate neurological deficits, and reduce mortality. Taken together, our results provided that enhancement of TRPML1 in neurons can prevent neuronal death induced by ischemia.

The ubiquitin-proteasome system (UPS) is one of the most important pathways responsible for cellular homeostasis [28]. Previous studies showed that the UPS plays a complex and unambiguous role in the pathophysiology of cerebral ischemia-reperfusion injury [29]. Our results showed the protein degradation of TRPML1 in the neurons after ischemic reperfusion injury. However, the mRNA level of TRPML1 in the primary cultured hippocampal neurons was not changed both 30 min and one hour after OGD. Therefore, the downregulation of TRPML1 occurred at posttranscriptional level. Additionally, we found that the degradation of TRPML1 was significantly suppressed after MG132 treatment, indicating the involvement of UPS. Evidence from a number of studies suggests that the important role of proteasome in ischemic reperfusion injury and proteasome inhibitors have been known to prevent brain ischemic damage, whose mechanisms are still largely unknown [30–32]. The ubiquitin E3 ligases, the critical components of this cascade, constitute the largest family of ubiquitin ligases with more than 600 predicted members. Zinc finger protein A20 was revealed as a ubiquitin ligase and attenuated the cerebral inflammatory injury in cerebral ischemia/reperfusion rats [33]. The HECT-domain E3 ligase Huwe1 (HECT, UBA, and WWE domain containing 1) involved in the ubiquitination, degradation of multiple proteins, was proven to interact with regulation of Gadd45 under oxygen-glucose deprivation and reperfusion injury in primary rat cortical neuronal cells [34]. Another ubiquitin E3 ligase TRAF6 was reported to be a key promoter of ischemic signaling cascades and neuronal death after cerebral ischemia reperfusion injury by ubiquitinating and activating Rac1 [35]. It is also reported that Siah1, parkin, and PARK2 act as an E3 ligase in cerebral ischemic reperfusion injury [36–40]. Therefore, identification of the E3 ligase of TRPML1 would further uncover the UPS function in cardiac arrest-induced ischemia.

ML-SA1, a structural analog of SF-51, can potently activate an inward rectifying TRPML1-dependent current similar to the one activated by phosphatidylinositol 3,5-bisphosphate (PI_3,5_P^2^) [41]. Recently ML-SA1 was identified to activate mammalian TRPML1 and shown to alleviate lipid accumulation in lysosomes of cellular models of lysosome storage diseases [42]. Enhanced TRPML1 triggers lysosomal Ca^2+^ release, autophagy induction, and lysosome biogenesis [17]. In the present study, we demonstrated that ML-SA1 could significantly promote the autophagy, attenuate the apoptosis, and alleviate neuron survival and neurological dysfunction that occur after ischemic reperfusion injury. Diverse neuroprotective materials have been suggested as agents for protecting the brain from global ischemic injury during cardiac arrest; however, their application for clinical treatment is not satisfactory. Our findings above indicated that TRPML1 could be a novel target for preventing and treating ischemic reperfusion brain injury.

Several limitations of this work should be noted. First, we tested only one dose of ML-SA1. The optimal timing and dosage of ML-SA1 should be fully considered in future studies. Second, we used an intracerebroventricular pretreatment model for ML-SA1 administration; however, additional studies are needed in posttreatment settings and dosing site.

## 5. Conclusion

Taken together, our study provides evidence that TRPML1 participates in delayed neuronal damage after TGI. In addition, our data demonstrate that ML-SA1 pretreatment attenuates neuron injury *in vivo* and *in vitro*. These protective effects result from enhancing autophagy, the inhibition of apoptosis, and protein ubiquitination. Thus, the regulation of TRPML1 could be a novel strategy for preventing and treating ischemic reperfusion brain injury.

## Data Availability

The data used to support the findings of this study are included within the article.
